# Development and validation of an automatic machine learning-based myocardial contusion prediction model in rib fracture patients: a retrospective multicenter cohort study

**DOI:** 10.3389/fmed.2026.1855699

**Published:** 2026-09-03

**Authors:** Yan Yang, Ping Liu, Ni Jia, Yisha Xu, Peng Wu, Zhigang Zhang, Tingyong Han, Yan Jiang, Xianpeng Huang, Long Li

**Affiliations:** 1 Department of Emergency Medicine, General Hospital of Western Theater Command, Chinese People's Liberation Army, Chengdu, Sichuan, China; 2 Emergency Department, Ya'an People's Hospital, Ya'an, Sichuan, China; 3 Emergency Department, Yucheng District People's Hospital of Ya'an, Ya'an, Sichuan, China; 4 Emergency Department, Mingshan District People's Hospital of Ya'an, Ya'an, Sichuan, China; 5 Emergency Department, Affiliated Hospital of Ya'an Polytechnic College, Ya'an, Sichuan, China; 6 Emergency Department, Ya'an Hospital of Traditional Chinese Medicine, Ya'an, Sichuan, China; 7 Department of General Medicine, Chinese People's Liberation Army Unit Hospital, Xi'an, Shanxi, China; 8 Department of Emergency and Critical Care Medicine, The 945th Hospital of Joint Logistics Support Force of Chinese People's Liberation Army, Ya'an, Sichuan, China

**Keywords:** automatic machine learning, clinical decision support system, explainable artificial intelligence, improved crested porcupine optimization algorithm, myocardial contusion, rib fracture

## Abstract

**Objective:**

This study aims to construct an automatic machine learning-based prediction model to accurately identify the risk of myocardial contusion in traumatic rib fracture patients, addressing the core limitations of insufficient sensitivity in traditional diagnostic methods and difficulties in clinical translation of existing prediction models.

**Methods:**

A multicenter retrospective study design was adopted, including 1,038 rib fracture patients from 8 hospitals (training set: 808 cases; testing set: 230 cases) from June 2024 to June 2025. An automatic machine learning framework (AutoML) was developed based on the Improved Crested Porcupine Optimization algorithm (ICPO). A dual-stage optimization process achieved key feature screening and hyperparameter tuning, with Synthetic Minority Over-sampling Technique (SMOTE) addressing class imbalance. Model performance was systematically evaluated using six core metrics (Sensitivity/SEN, Specificity/SPE, F1-score, AUC, etc.), calibration curves, and Decision Curve Analysis (DCA). Feature robustness was validated via LASSO regression, and SHAP interpretability models analyzed key variable contributions. A clinical decision support interactive tool was developed on the MATLAB platform.

**Results:**

The AutoML model demonstrated superior performance on the testing set (classification threshold = 0.5; TP = 138, TN = 64, FP = 19, FN = 9; PRE = 0.8790, SEN = 0.9388, SPE = 0.7711, ACC = 0.8783, F1 = 0.9079, AUC = 0.9537), significantly outperforming traditional models such as logistic regression and support vector machine. SHAP analysis identified sternal fracture, anterolateral upper thoracic fracture, pneumomediastinum, and ISS score as key contributing features in the predictive model. SHAP analysis further suggested potential interaction patterns between pneumomediastinum and ISS > 20, and between sternal fracture and pneumothorax within the model.

**Conclusion:**

The AutoML prediction model exhibits high accuracy and strong interpretability. Its integrated visual interactive tool represents an initial step toward addressing clinical translation barriers, providing intelligent decision support for early warning and stratified intervention of myocardial contusion in rib fracture patients.

## 1 Introduction

Rib fracture is a clinically common type of trauma, often caused by high-impact forces such as traffic accidents or falls from height. This high-energy blunt chest trauma not only damages skeletal structures but is frequently complicated by severe intrathoracic organ injuries, among which concomitant myocardial contusion (MC) warrants particular vigilance (1). Although myocardial contusion may occasionally present with occult clinical symptoms, it remains a key factor contributing significantly to increased morbidity and mortality following blunt chest trauma (2). Its associated complications—fatal arrhythmias, cardiogenic shock, and cardiac rupture—pose a grave threat to patient survival and constitute a critical clinical challenge requiring urgent resolution in trauma emergency and intensive care settings (3). Early and accurate identification of high-risk myocardial contusion patients is fundamental to implementing timely interventions and improving prognoses. However, current diagnostic methods face significant hurdles: traditional reliance on electrocardiographic changes (e.g., ST-T abnormalities, arrhythmias) and elevated serological cardiac markers such as creatine kinase isoenzyme (CK-MB) and troponin I/T (cTnI/cTnT), while indicative, suffer from limitations in diagnostic efficacy—insufficient sensitivity and specificity, coupled with an inability to clearly delineate injury severity or dynamic progression trends (4–8). Trauma severity scoring systems (e.g., ISS, NISS, c-AIS), although reflective of overall injury burden, mainly serve comprehensive evaluation of multisystem trauma. Their capability for specific prediction of myocardial injury is limited, failing to precisely quantify individualized risks of myocardial contusion. Compounding this complexity is the fact that rib fracture patients often develop multiple intrathoracic complications such as pneumothorax, hemothorax, and pulmonary contusion. These comorbidities themselves can elicit non-specific symptoms like chest pain and dyspnea, further confounding the clinical identification of myocardial contusion and escalating diagnostic difficulty (9, 10).

In recent years, with the rapid advancement of artificial intelligence (AI) and machine learning (ML) technologies, data-driven approaches for constructing clinical disease prediction models have demonstrated immense potential, offering novel insights for accurately assessing myocardial contusion risk. Both domestic and international studies have explored risk prediction models for trauma contexts, with some achieving modest success in predictive performance. However, in-depth analysis reveals three major limitations in previous research (11–14). First, there exist significant flaws in model architecture: most existing models are based on single algorithms or relatively simplistic traditional models, rendering them inadequate in adapting to complex trauma data, capturing nonlinear relationships, and uncovering deep variable interactions. Particularly when confronted with the highly multidimensional and potentially intricate data—encompassing multi-system injury indicators, vital sign fluctuations, detailed anatomical localization, and dynamic laboratory biochemical markers—these models often struggle to discern true critical signals from noise, resulting in prediction accuracy insufficient for high-stakes clinical decision-making. Second, inadequate model reliability and interpretability abound: a substantial portion of prior models focus primarily on outputting predictions while neglecting to effectively elucidate the model’s internal decision logic and evidential basis. Clinicians confronted with “black-box” predictions cannot trace or comprehend how key variables influence predicted probabilities or their contribution weights, drastically diminishing model credibility and physician acceptance. The lack of robust interpretability analysis constitutes a bottleneck for clinical endorsement and real-world deployment of novel models. Third, barriers to clinical translation are prohibitively high: most previously developed prediction models remain confined to the research-validation stage and fail to transform into practical, user-friendly tools for frontline clinicians. Promoting and implementing existing approaches typically demands users to possess programming skills, algorithmic knowledge, or specialized statistical expertise to run and interpret results. This technical gap clashes starkly with the urgent, high-efficiency demands of clinical practice, directly hindering the adoption, widespread use, and practical value realization of AI-assisted decision systems in primary care institutions or busy emergency trauma centers. These gaps and inadequacies constitute critical vulnerabilities along the research-to-application pipeline for myocardial contusion risk prediction in rib fracture patients.

Driven by a profound understanding of these technological limitations and clinical translation gaps in prior studies, this research aims to leverage high-quality, multicenter clinical datasets to develop and refine an automatic machine learning prediction model capable of sensitively capturing myocardial contusion risk in rib fracture patients. Such a model will assist clinicians in making timelier and more precise individualized decisions, thereby contributing crucially to improving the overall prognosis of patients with rib fractures complicated by myocardial contusion, and enhancing the quality and efficiency of trauma care comprehensively.

## 2 Methods

### 2.1 Study subjects and data collection

This study employed a multicenter retrospective observational cohort design, systematically collecting patients with traumatic rib fractures admitted to eight hospitals between June 2024 and June 2025. A total of 1,038 subjects were included based on predefined criteria. As a retrospective study, it was exempt from patient informed consent and received ethical approval from the Ethics Committee of the General Hospital of Western Theater Command (Approval No.: 2025–091132), adhering to the Declaration of Helsinki standards.

Inclusion criteria: (1) Traumatic rib fractures confirmed by CT or X-ray; (2) Time from injury to admission <24 h; (3) Age ≥18 years; (4) Completion of electrocardiogram (ECG) and cardiac biomarker testing within 24 h of admission; (5) Absence of severe immune deficiency or drug abuse.

Exclusion criteria: (1) Multiple organ dysfunction syndrome; (2) Coagulation disorders or oral anticoagulant use; (3) Emergency thoracotomy; (4) Severe cognitive or communication impairment; (5) Myocardial infarction or myocarditis within 2 weeks; (6) Alcoholism or abnormal liver function before admission; (7) Incomplete medical records (>10% missing variables).

Data collection: all data were extracted from hospital electronic medical record (EMR) systems using structured forms and independently verified by two certified researchers. Collected parameters included: (1) Demographics: gender, age, comorbidities (hypertension, diabetes, COPD, hyperlipidemia); (2) Vital signs on admission: systolic blood pressure, diastolic blood pressure, heart rate; (3) Fracture characteristics: Type: Bilateral fractures, flail chest; Number of fractures (continuous variable); Location (12-segment division): Upper chest: Sternal-proximal segment, anterolateral segment, posterolateral segment, spinal-proximal segment; Mid-chest: Sternal-proximal segment, anterolateral segment, posterolateral segment, spinal-proximal segment; Lower chest: Sternal-proximal segment, anterolateral segment, posterolateral segment, spinal-proximal segment; (4) Concomitant thoracic injuries: Pneumothorax, pulmonary contusion, pneumomediastinum, hemothorax, sternal fracture; (5) Trauma scores: Thoracic Abbreviated Injury Scale (c-AIS), Injury Severity Score (ISS), New Injury Severity Score (NISS). It should be noted that, to avoid circular reasoning, when computing the trauma severity scores (ISS, NISS, c-AIS), we explicitly excluded the cardiac anatomic AIS code directly attributable to myocardial contusion. The final ISS and NISS calculations incorporated only the severity codes for concomitant intrathoracic injuries (e.g., rib fractures, pulmonary contusion, pneumothorax, hemothorax), while the anatomic rating of the myocardial injury itself was omitted. Consequently, the trauma scores presented in this study reflect strictly non-cardiac injury burden, and they are entirely independent of the electrocardiographic and myocardial enzyme changes used to define the outcome variable; (6) Laboratory indices: inflammatory markers: White blood cell count (WBC), C-reactive protein (CRP); Metabolic markers: total cholesterol (TC), triglycerides (TG), high-density lipoprotein (HDL), low-density lipoprotein (LDL); Hepatic/renal function: Albumin (ALB), total bilirubin (TBIL), aspartate aminotransferase (AST), alanine aminotransferase (ALT), blood urea nitrogen (BUN), creatinine (Cr); Hematological indices: Hemoglobin (Hb), hematocrit (HCT), platelet count (PLT); (7) Outcome variable: myocardial contusion (Yes/No), diagnosed through a composite reference standard combining (15): the primary outcome was myocardial contusion, diagnosed using a hierarchical composite reference standard. Definite myocardial contusion was defined by imaging evidence of myocardial injury on contrast-enhanced cardiac MRI (late gadolinium enhancement, myocardial edema on T2-weighted imaging, or regional wall motion abnormality) or, when MRI was unavailable, on transthoracic echocardiography demonstrating new regional wall motion abnormalities. Probable myocardial contusion, in patients without confirmatory advanced imaging, was defined by the concurrent presence of (a) new and significant ECG abnormalities (ST-segment deviation ≥1 mm in two contiguous leads, T-wave inversion, or new-onset clinically significant arrhythmia) AND (b) elevated hs-cTnI >99th percentile upper reference limit with a dynamic rising and/or falling pattern on serial measurements (at least two measurements within 24 h). Patients with isolated ECG changes or isolated troponin elevation in the absence of imaging confirmation were classified as non-contusion cases. This definition was established to maximize specificity for clinically significant myocardial injury. Two investigators independently adjudicated the outcome, and disagreements were resolved by a third physician. Since transthoracic echocardiography and cardiac MRI were not routinely performed for all patients, verification bias was unavoidable. To this end, we report the number of definite and probable cases, the completion status of each diagnostic examination, and conducted a sensitivity analysis restricted to imaging-confirmed cases only.

### 2.2 Automatic machine learning model construction

**Figure 1:**
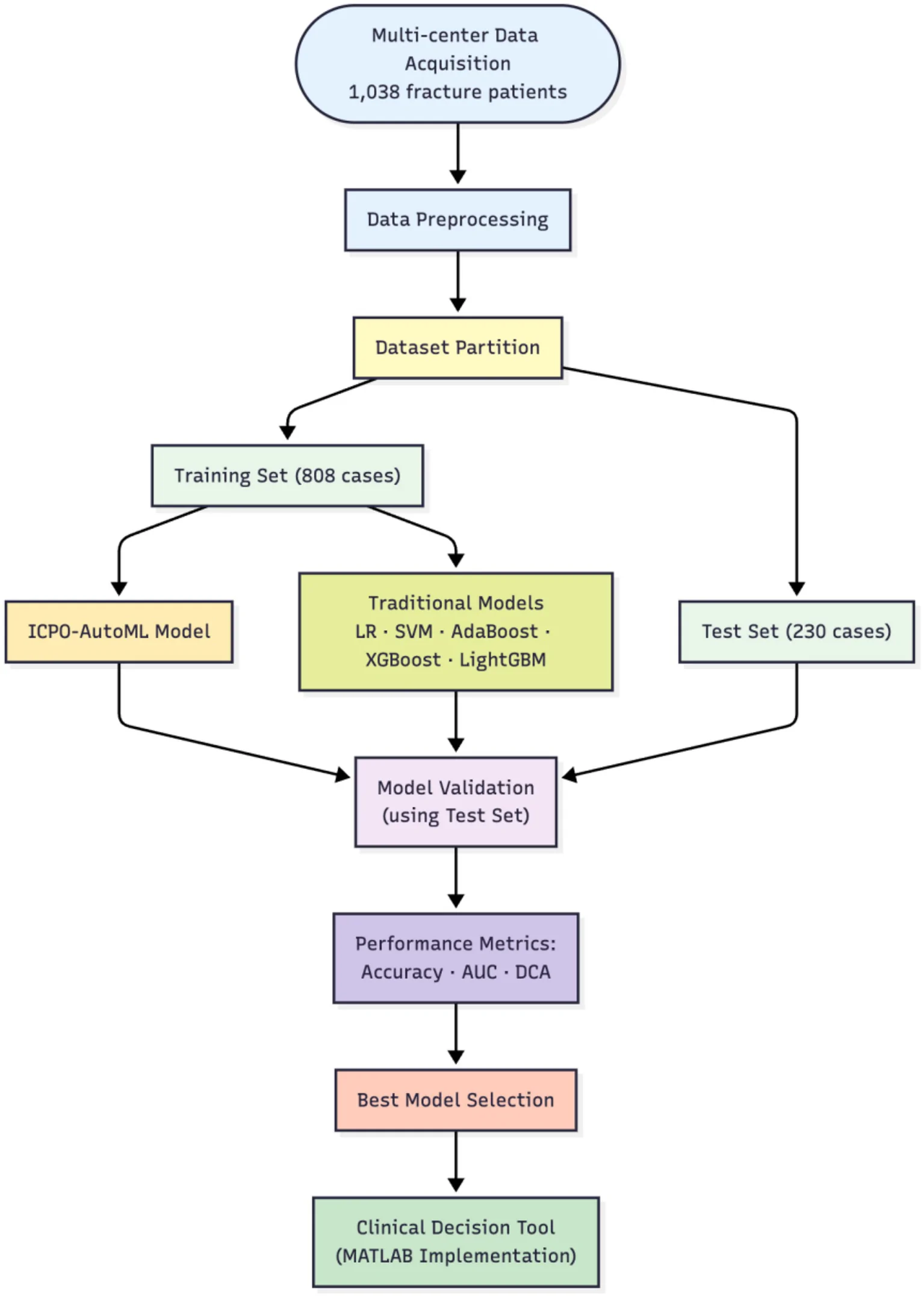
Study flow chart.

Among the 1,038 included patients, 808 patients from the General Hospital of Western Theater Command, the 945th Hospital of Joint Logistics Support Force of the Chinese People’s Liberation Army, Ya’an People’s Hospital, Yucheng District People’s Hospital, and Mingshan District People’s Hospital constituted the training set, and the remaining 230 patients constituted the independent testing set. All models were implemented on the MATLAB 2024b platform and followed a rigorous standardized preprocessing and modeling pipeline, with the core principle of constraining all data operations within the training set to eliminate any form of information leakage. To ensure a fair comparison, logistic regression (LR), support vector machine (SVM), AdaBoost, XGBoost, LightGBM, and the proposed AutoML model were trained and evaluated under an identical pipeline, including the same training/testing split, the same nested cross-validation folds, the same preprocessing steps, the same optimization metric, the same computational budget for hyperparameter search, and the same independent testing set. The detailed study flowchart is shown in Figure 1.

We proposed an automatic machine learning framework (AutoML) based on the Improved Crested Porcupine Optimization (ICPO) to simultaneously achieve key feature selection and hyperparameter optimization for myocardial contusion prediction. The Crested Porcupine Optimization (CPO) is a novel swarm intelligence optimization algorithm inspired by the collective behavior of crested porcupines. This algorithm solves complex optimization problems by simulating the collective intelligence exhibited by crested porcupines during foraging and predator avoidance. CPO was improved into ICPO: chaotic mapping was adopted to optimize the initial population distribution to enhance spatial ergodicity, combined with a dynamic Lévy flight step-size strategy to balance the search efficiency between exploration and exploitation phases. To further validate the performance of ICPO, the CEC2022 benchmark test functions were used to evaluate algorithm performance. To further validate the performance of ICPO, this study included the Whale Optimization Algorithm (WOA), Gray Wolf Optimizer (GWO), Particle Swarm Optimization (PSO), Genetic Algorithm (GA), and two hybrid algorithms (GA-PSO, GA-ACO) for comparison. In this study, progressive data perturbations were artificially injected to simulate measurement errors and missing values in real clinical data, with perturbation levels sequentially set at 0–10%, 10–20%, 20–30%, and 30–40%. Under each perturbation level, each algorithm was independently trained 30 times to calculate performance statistics. Meanwhile, a feature selection module was designed to evaluate the model’s complexity control ability. Feature selection stability was expressed as the selection probability, defined as the frequency with which each feature was selected by ICPO across 100 independent nested cross-validation/optimization runs. This calculation was accomplished on the training set only and was performed prior to SMOTE to avoid the influence of oversampling on feature selection frequency.

To uphold the principle of preventing data leakage, we designed a nested cross-validation architecture. Throughout the entire modeling process, the independent testing set was completely isolated and used only for the final model performance evaluation. Within the five-fold cross-validation of the training set, the following steps were strictly executed within each fold: first, the internal training set and internal validation set of that fold were separated; then, the Synthetic Minority Over-sampling Technique (SMOTE) was applied only to the internal training set, while the internal validation set and the independent testing set remained in their original class distributions without any resampling. Although myocardial contusion was the majority class in both the training set (460/808, 56.93%) and the testing set (147/230, 63.91%), the non-contusion class remained the minority class (348/808, 43.07% in training; 83/230, 36.09% in testing). Accordingly, SMOTE was used to oversample the minority non-contusion class rather than the contusion class, in order to mitigate majority-class bias toward myocardial contusion that would otherwise inflate sensitivity at the expense of specificity and probability calibration. After that, the optimization process of the ICPO algorithm was initiated on the SMOTE-processed internal training set, whereas the fitness function was evaluated exclusively on the untouched internal validation set. Because SMOTE alters the training-class prior and can shift predicted probabilities, Platt scaling was fitted on the non-SMOTE internal validation fold and then fixed for application to the independent testing set. As a sensitivity check, all models were also retrained without SMOTE under otherwise identical feature sets, hyperparameter search spaces, and evaluation procedures. The same fold assignments were shared across all candidate models so that differences in performance could not be attributed to unequal data partitions.

Regarding missing data, the inclusion criteria of this study explicitly required “complete case data (key variable missingness not exceeding 10%),” thus initially ensuring the overall data quality. For the small amount of missing values still sporadically present in the finally included samples, we did not use simple imputation methods that could cause information leakage or introduce bias (e.g., mean or median imputation). We adopted the Multiple Imputation by Chained Equations (MICE) method, which possesses excellent statistical properties under the missing at random assumption. The construction of the imputation model strictly followed the principle of “fitting only within the training set”: we first established the imputation model and learned its parameters using the training set data, and then applied this model to impute missing values in both the training set and the testing set, thus completely isolating the testing set data from influencing the imputation process. All continuous variables were standardized using Z-score normalization before modeling, and the calculation of the mean and standard deviation was entirely based on the training set data; the testing set was transformed using the parameters derived from the training set.

All comparator models and the AutoML model were optimized under the same nested cross-validation scheme and the same fitness metric (mean ROC-AUC on the untouched internal validation fold). Specifically, LR, SVM, AdaBoost, XGBoost, LightGBM, and AutoML used identical outer/inner fold indices, identical MICE imputation and Z-score parameters derived from the training partition only, and identical SMOTE handling restricted to the minority non-contusion class within each inner training fold. Hyperparameters for every model were tuned by ICPO within predefined search spaces and under the same computational budget (same population size and maximum number of iterations). The search spaces were as follows: for LR, L2-regularized logistic regression with regularization strength C in [0.01, 100]; for SVM, an RBF kernel with penalty C and kernel coefficient gamma in [2^−8, 2^8]; for AdaBoost, learning rate and number of estimators; and for XGBoost and LightGBM, learning rate, maximum depth, and subsample ratio within predefined continuous ranges. AutoML differed only in that ICPO jointly searched the feature subset and hyperparameters, whereas comparator models used the same candidate feature pool without an additional model-specific preprocessing shortcut. After model selection, all models were refit on the full training set with the selected settings and evaluated once on the locked independent testing set. The implementation flow is provided in Supplementary material 1.

For reproducibility, all stochastic procedures used a fixed random seed of 2024, including SMOTE, model initialization, and bootstrap resampling. Binary clinical variables were coded as 0/1; continuous variables were retained as numeric values and standardized using training-set means and standard deviations; trauma scores were treated as continuous features; and the outcome was coded as myocardial contusion = 1 versus non-contusion = 0. Remaining missing values after enrollment screening were handled by MICE fitted on the training set only. The final hyperparameters selected for each model were as follows: LR (L2, C = 1.74); SVM (RBF, C = 4.00, gamma = 0.03125); AdaBoost (learning rate = 0.35, n_estimators = 120); XGBoost (learning rate = 0.048, max_depth = 4, n_estimators = 280); LightGBM (learning rate = 0.042, max_depth = 5, n_estimators = 320); and AutoML (learning rate = 0.037, max_depth = 4, n_estimators = 360), with a classification threshold of 0.5. The complete MATLAB analysis script is provided as Supplementary material 3.

### 2.3 Evaluation metrics

A multidimensional evaluation system was established: (1) Classification performance: For the predictive model, six core metrics were adopted to systematically assess discriminative ability and stability in class-imbalanced scenarios: Accuracy (ACC), Sensitivity (SEN), Specificity (SPE), Precision (PRE), F1 score (harmonic mean of precision and recall), Area Under the ROC Curve (ROC-AUC), and Area Under the Precision-Recall Curve (PR-AUC). (2) Calibration performance: Calibration curves combined with the Brier score (lower scores indicate higher prediction accuracy) were used to evaluate probability prediction precision. (3) Clinical application: Decision Curve Analysis (DCA) was applied to quantify clinical utility by calculating the net benefit (NB) at different threshold probabilities:


$$\mathrm{NB}=\frac{\mathrm{TP}}{N}-\frac{\mathrm{FP}}{N}\times \frac{p_{t}}{1-p_{t}}$$

where *TP* is true positives, *FP* is false positives, *N* is total sample size, and pt. is the risk threshold. By comparing *NB* with reference lines for traditional intervention strategies, the effective interval for model-assisted decision-making was validated.

To provide a formal statistical comparison of discriminative ability among models, this study employed the DeLong test to evaluate the differences in the area under the receiver operating characteristic curve (ROC-AUC) between different models. The DeLong test constructs a variance estimate of the AUC difference, fully accounting for the paired correlation arising from models being evaluated on the same testing set, and is the most widely used and robust parametric method for paired AUC comparisons. We calculated the 95% confidence intervals of the AUC difference (ΔAUC) between the AutoML and each comparison model; these intervals were obtained based on the DeLong variance estimate via normal approximation and directly correspond to the significance determination of the test. Meanwhile, to assess the uncertainty of the generalization performance of a single model, we calculated the two-sided 95% confidence intervals of ROC-AUC, sensitivity, specificity, and F1-score for all models on the testing set based on 2,000 stratified bootstrap resampling iterations. Statistical comparison of calibration was accomplished through the Brier score and its bootstrap 95% confidence intervals. Unless otherwise specified, threshold-dependent metrics (SEN, SPE, PRE, ACC, and F1 score) were calculated at a probability cut-off of 0.5, with the corresponding confusion matrix counts (TP, TN, FP, and FN) reported concurrently.

### 2.4 Interpretability analysis

After initial screening of prognostic prediction features via the AutoML framework, Lasso regression analysis was applied to verify feature robustness, followed by SHAP interpretability modeling to analyze clinical rationality. The specific workflow encompassed: (1) AutoML feature preliminary screening: Based on predefined search spaces and optimization objectives, the AutoML algorithm automatically identified feature subsets significantly associated with prognosis; (2) Lasso feature validation: After the AutoML/ICPO framework completed core feature selection and model development, this study further employed LASSO regression as an independent, complementary concordance analysis to evaluate the stability of the AutoML-selected variables within an alternative regularized modeling approach. It is important to emphasize that the LASSO analysis and the AutoML model development pipeline were strictly isolated: the LASSO results did not participate in AutoML feature selection, model training, hyperparameter optimization or final prediction model variable determination, nor did they feed back to influence the AutoML model performance evaluation. The sole purpose was to examine whether the core features selected by AutoML could also be supported within an L1 regularization framework. Subsequently, SHAP-based interpretability analysis was conducted on the final AutoML model to quantify the contribution of each core feature to the model’s predictions; (3) SHAP (Shapley Additive Explanations) interpretability analysis: Based on game theory, the SHAP algorithm quantified feature contributions, revealing the overall influence intensity of key variables through global feature importance ranking and enabling visualization of prediction logic to verify rationality.

### 2.5 Clinical decision system

In our study, MATLAB’s App Designer function was utilized to develop a clinical decision support software. This software integrates the constructed predictive model to provide clinicians with an intuitive, user-friendly tool for prognosis assessment in patients. The software is web-deployable for convenient clinical use.

### 2.6 Statistical methods

All study data were uniformly imported into the SPSS 26.0 statistical analysis platform for standardized processing. Continuous variables with normal distribution were expressed as mean ± standard deviation (x̄ ± s), while those with non-normal distribution were denoted as median (interquartile range) (M [IQR]); categorical variables were expressed as frequency and percentage (*n* (%)). For inter-group comparisons, continuous variables first underwent normality testing: if both groups conformed to normal distribution, univariate *t*-test was applied; otherwise, Mann–Whitney U test was used. Categorical variables were compared using Pearson’s chi-square test. Statistical significance was determined based on *p*-values (two-tailed test, significance threshold *α* = 0.05), and results were presented in tabular formats.

## 3 Results

### 3.1 Characteristics of the study cohort

The overall cohort had a mean age of 56.55 ± 12.95 years, comprising 720 males (69.36%) and 318 females (30.64%). The training set (*n* = 808) and testing set (*n* = 230) showed no statistically significant differences in baseline characteristics or laboratory indices (all *p* > 0.05). Myocardial contusion incidence was highly consistent between groups (training set: 56.93% vs. testing set: 63.91%; *χ*^2^ = 3.595, *p* = 0.058). Details are presented in Table 1.

**Table 1:** Comparison of clinical characteristics between Training Set and Testing Set.

Feature	Training set (*n* = 808)	Testing set (*n* = 230)	Statistic	*p*-value
Outcome indicator				
Myocardial contusion (MC), *n* (%)	460 (56.93)	147 (63.91)	3.595	0.058
Demographic characteristics				
Age (years), *Mean* ± *SD*	56.16 ± 13.15	57.91 ± 12.16	1.142	0.254
Male, *n* (%)	562 (69.55)	158 (68.70)	0.062	0.803
Hypertension, *n* (%)	312 (38.61)	92 (40.00)	0.145	0.704
Diabetes, *n* (%)	122 (15.10)	31 (13.48)	0.374	0.541
COPD, *n* (%)	86 (10.64)	25 (10.87)	0.010	0.922
Hyperlipidemia, *n* (%)	121 (14.98)	36 (15.65)	0.064	0.8
Vital signs				
Systolic blood pressure (mmHg), *M*[*Q_1_*, *Q_3_*]	134 [123, 148]	135 [124, 150]	0.857	0.391
Diastolic blood pressure(mmHg), *M*[*Q_1_*, *Q_3_*]	79 [74, 88]	80 [75, 88]	0.732	0.464
Heart rate (beats/min), *M* [*Q_1_*, *Q_3_*]	78 [72, 85]	79 [73, 86]	1.025	0.305
Fracture characteristics				
Bilateral rib fracture, *n* (%)	298 (36.88)	90 (39.13)	0.387	0.534
Flail chest, *n* (%)	185 (22.90)	54 (23.48)	0.034	0.853
Number of fractures (count), *M* [*Q_1_*, *Q_3_*]	5 [3, 7]	5 [3, 7]	0.318	0.75
Fracture location, *n* (%)				
Sternal-proximal segment, upper chest	142 (17.57)	40 (17.39)	0.004	0.949
Anterolateral segment, upper chest	336 (41.58)	94 (40.87)	0.038	0.846
Posterolateral segment, upper chest	103 (12.75)	28 (12.17)	0.053	0.817
Spinal-proximal segment, upper chest	98 (12.13)	29 (12.61)	0.038	0.845
Sternal-proximal segment, mid-chest	127 (15.72)	35 (15.22)	0.034	0.854
Anterolateral segment, mid-chest	318 (39.36)	91 (39.57)	0.003	0.954
Posterolateral segment, mid-chest	135 (16.71)	37 (16.09)	0.050	0.823
Spinal-proximal segment, mid-chest	240 (29.70)	60 (26.09)	1.139	0.286
Sternal-proximal segment, lower chest	86 (10.64)	23 (10.00)	0.079	0.779
Anterolateral segment, lower chest	287 (35.52)	81 (35.22)	0.007	0.933
Posterolateral segment, lower chest	132 (16.34)	39 (16.96)	0.050	0.823
Spinal-proximal segment, lower chest	94 (11.63)	28 (12.17)	0.050	0.822
Thoracic injuries, *n* (%)				
Pneumothorax	370 (45.79)	108 (46.96)	0.098	0.755
Pulmonary contusion	562 (69.55)	158 (68.70)	0.062	0.803
Pneumomediastinum	48 (5.94)	13 (5.65)	0.027	0.87
Hemothorax	288 (35.64)	79 (34.35)	0.132	0.717
Sternal fracture	45 (5.57)	13 (5.65)	0.002	0.961
Trauma scores, *M* [*Q_1_*, *Q_3_*]				
c-AIS	3 [3, 4]	3 [3, 4]	0.807	0.42
ISS	16 [9, 21]	16 [10, 22]	1.105	0.269
NISS	27 [18, 34]	26 [17, 33]	0.942	0.346
Laboratory indices, *M* [*Q_1_*, *Q_3_*]				
WBC(×10^9^/L)	8.9 [7.2, 11.5]	8.8 [7.1, 11.3]	0.732	0.464
Hb(g/L)	130 [121, 142]	129 [120, 141]	1.048	0.295
PLT(×10^9^/L)	180 [145, 215]	178 [142, 212]	0.826	0.409
HCT(%)	0.40 [0.37, 0.43]	0.40 [0.37, 0.43]	0.215	0.83
TC(mmol/L)	4.3 [3.8, 4.9]	4.3 [3.9, 4.8]	0.328	0.743
TG(mmol/L)	1.1 [0.8, 1.4]	1.1 [0.8, 1.5]	0.647	0.517
HDL(mmol/L)	1.3 [1.1, 1.5]	1.3 [1.1, 1.5]	0.238	0.812
LDL(mmol/L)	2.5 [2.1, 3.0]	2.5 [2.1, 2.9]	0.752	0.452
ALB(g/L)	41.5 [38.5, 43.8]	41.3 [38.3, 43.6]	0.986	0.324
TBIL(μmol/L)	13.8 [10.3, 18.5]	13.5 [10.1, 18.2]	0.528	0.597
AST(U/L)	29 [23, 42]	30 [23, 43]	0.875	0.381
ALT(U/L)	25 [18, 38]	26 [19, 39]	0.912	0.362
BUN(mmol/L)	5.4 [4.5, 6.3]	5.3 [4.4, 6.2]	0.728	0.467
Cr(μmol/L)	70 [58, 82]	69 [57, 81]	0.854	0.393

### 3.2 Algorithm optimization performance

**Figure 2:**
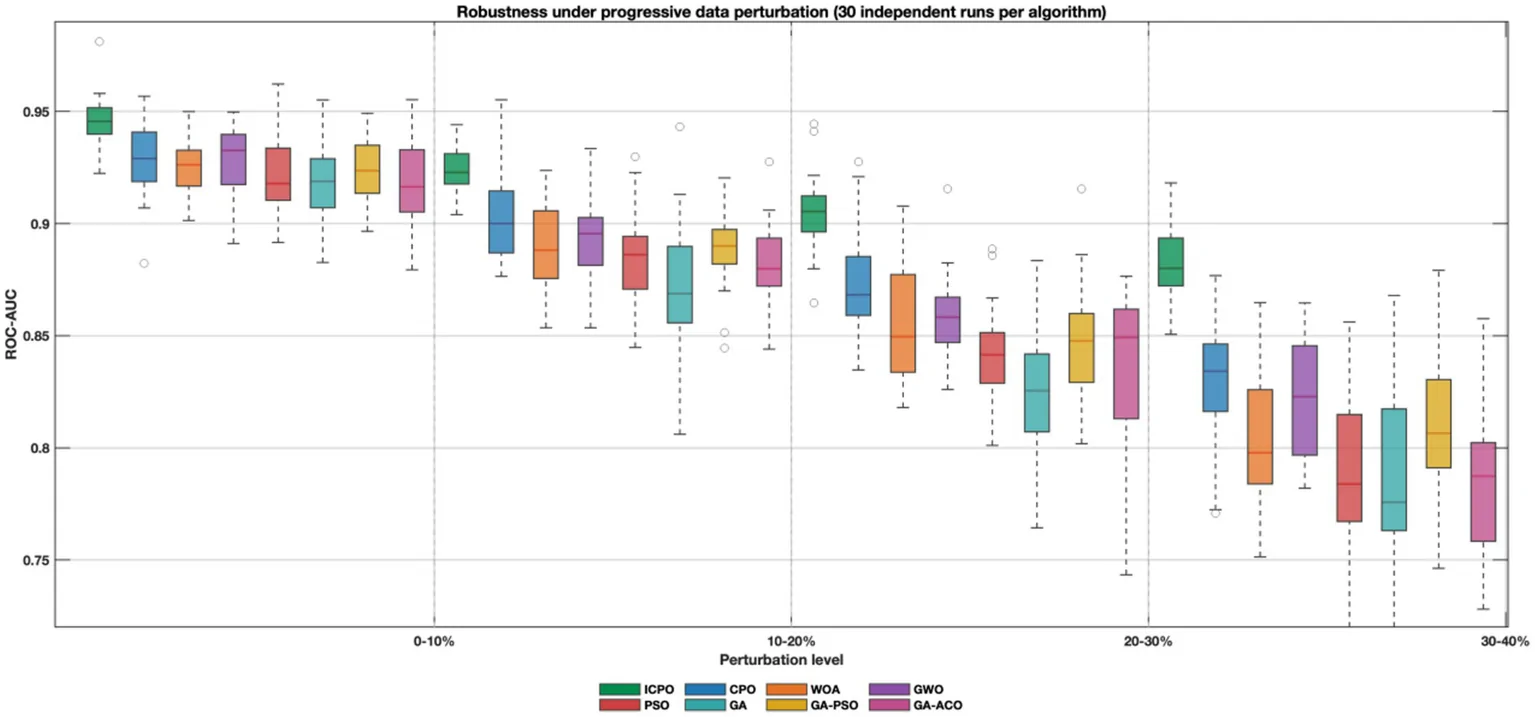
Robustness comparison of intelligent algorithm predictions. This figure shows the stability performance of eight intelligent algorithms under increasing data perturbations. Box plots represent the distribution of cross-validated AUC values from 30 repeated experiments.

The simulation test results of standard benchmark functions for swarm intelligence algorithms are presented in Supplementary material 2, showing that the ICPO algorithm had significant advantages in global optimization performance and convergence efficiency. By artificially injecting progressive data perturbations (four levels from 0–10% to 30–40%), this study employed box plots to evaluate the noise resistance performance of each algorithm (Figure 2). Under low perturbation conditions (0–10%), the average predictive accuracy of the eight algorithms was AUC = 0.926; under high perturbation conditions (30–40%), model performance declined to an average AUC = 0.813, a relative decrease of 12.2%. Among them, the ICPO algorithm exhibited the best stability: its predictive accuracy decreased from AUC = 0.945 in low perturbation scenarios to AUC = 0.883 in high perturbation scenarios, and the relative decrease rate (6.6%) was significantly lower than that of the baseline CPO group (decrease rate 10.6%), while maintaining the highest absolute predictive accuracy across all four noise levels. The ICPO maintained the highest predictive performance across all noise levels, indicating its stronger noise resistance capability in real clinical data.

**Figure 3:**
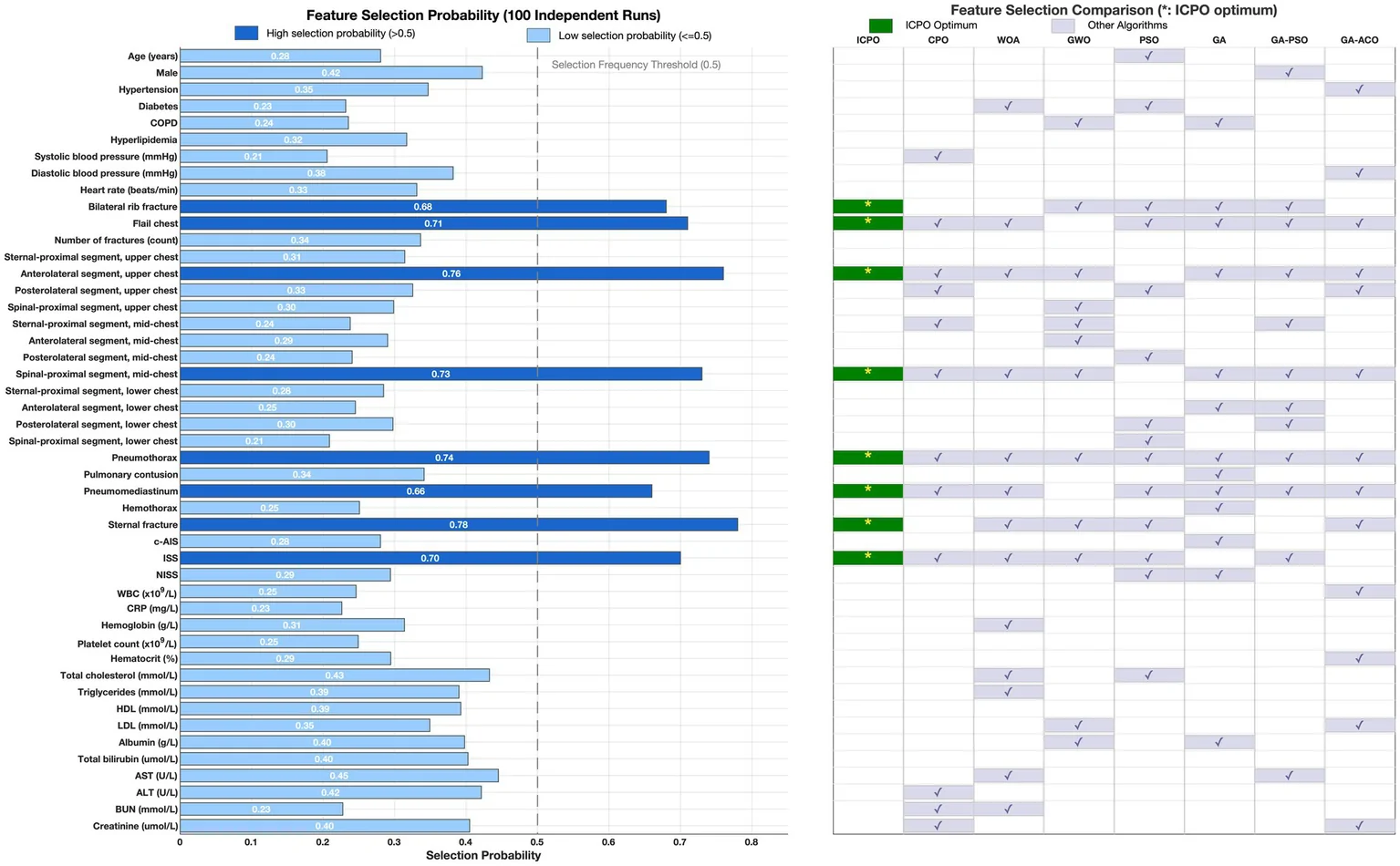
Clinical feature analysis and feature selection optimization. The left panel shows the selection probability of each clinical feature across 100 independent ICPO training runs, with dark blue indicating high selection probability (>0.5), light blue indicating low selection probability (≤0.5), and the gray dashed line representing the 0.5 threshold. The right panel shows the feature selection results of each algorithm, with dark green squares denoting features ultimately selected by ICPO, and light purple squares denoting features selected by other algorithms. The above selection probabilities were calculated based on the training set (*n* = 808) and completed prior to SMOTE oversampling, and they do not represent univariate correlation coefficients between features and the outcome.

Among the 47 candidate features, 8 features had a selection probability greater than 0.5 across the 100 independent training runs, namely sternal fracture (*r* = 0.78), anterolateral fracture upper chest (*r* = 0.76), pneumothorax (*r* = 0.74), paraspinal fracture mid chest (*r* = 0.73), flail chest (*r* = 0.71), ISS score (*r* = 0.70), bilateral rib fractures (*r* = 0.68), and pneumomediastinum (*r* = 0.66). The ICPO algorithm demonstrated excellent feature reduction capability, stably selecting 8 high-correlation predictors (mean correlation coefficient *r* = 0.72, SD = 0.04), representing a 37% reduction in features compared to the average of 12.7 features selected by other algorithms. This feature refinement mechanism effectively reduced model complexity while maintaining a high average correlation (Figure 3).

### 3.3 Myocardial contusion prediction model performance

**Figure 4:**
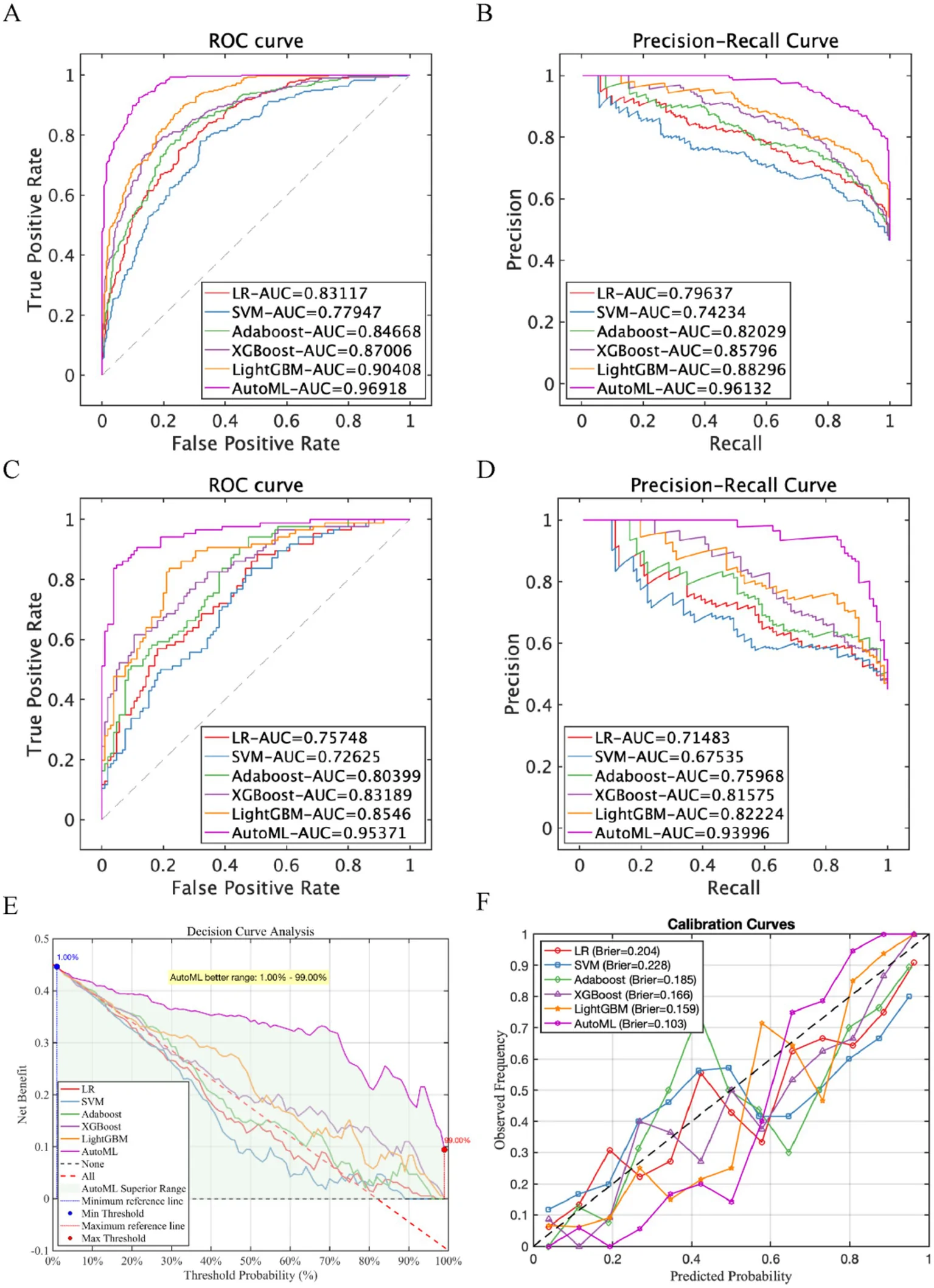
Predictive model training and performance validation. **(A)** Training set ROC curve; **(B)** Training set PR curve; **(C)** Testing set ROC curve; **(D)** Testing set PR curve; **(E)** Testing set DCA curve; **(F)** Testing set calibration curve.

**Figure 5:**
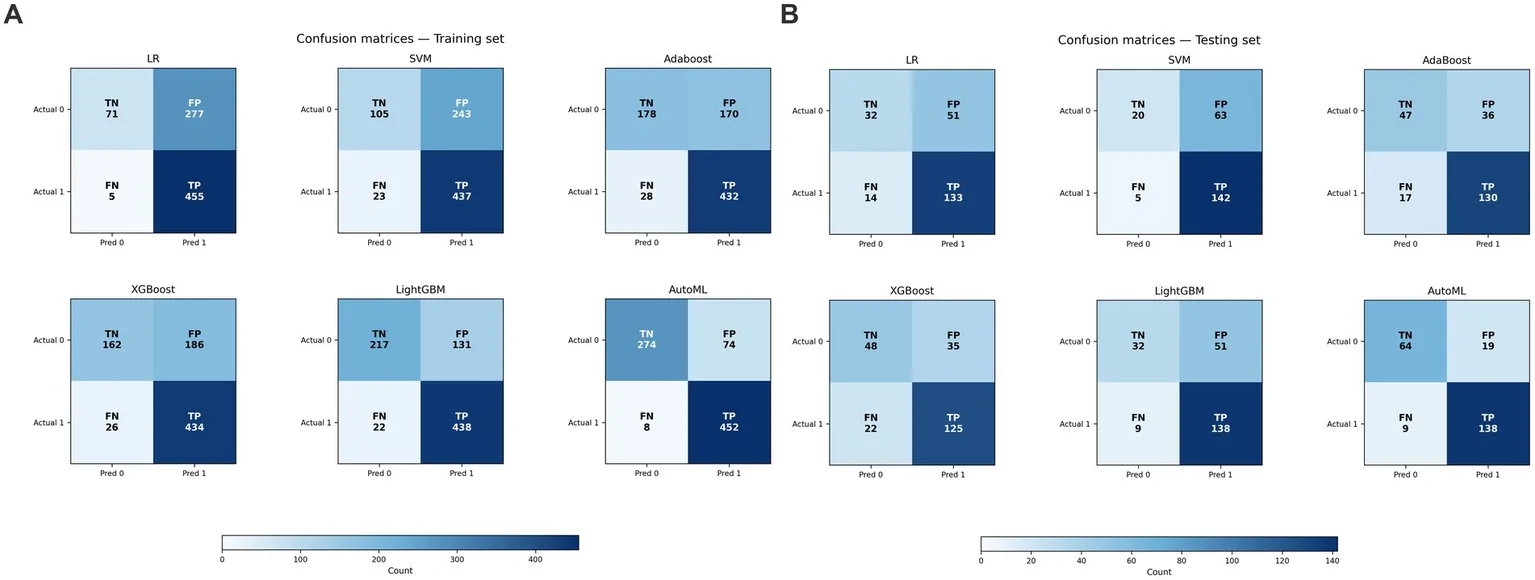
Confusion matrix of the prediction results. **(A)** Training set; **(B)** Testing set.

In the training set, the performance of the six machine learning models is shown in Table 2 and Figures 4A,B. AutoML achieved the optimal overall performance, with an ROC-AUC of 0.9692 and a PR-AUC of 0.9613, along with an F1-score of 0.8818, demonstrating an excellent balance between recall and precision. The confusion matrices of the prediction results for the training set and testing set are shown in Figure 5.

**Table 2:** Predictive performance of each model in the training set.

Models	PRE	SEN	SPE	ACC	F1	ROC-AUC	PR-AUC
LR	0.6216	0.9891	0.2040	0.6510	0.7634	0.8312	0.7964
SVM	0.6426	0.9500	0.3017	0.6708	0.7667	0.7795	0.7423
Adaboost	0.7176	0.9391	0.5115	0.7550	0.8136	0.8467	0.8203
XGBoost	0.7000	0.9435	0.4655	0.7376	0.8037	0.8701	0.8580
LightGBM	0.7698	0.9522	0.6236	0.8106	0.8513	0.9041	0.8830
AutoML	**0.8593**	**0.9826**	**0.7874**	**0.8985**	**0.9168**	**0.9692**	**0.9613**

Bold data indicate optimal results.

All models were compared under identical folds, preprocessing, optimization metric, computational budget, and testing data. The discriminative performance of all models on the independent testing set and the formal DeLong test comparisons with AutoML are summarized in Tables 3, 4, and Figures 4C,D. AutoML maintained excellent generalization ability on the independent testing set. At a classification threshold of 0.5, the confusion matrix showed TP = 138, TN = 64, FP = 19, FN = 9; the ROC-AUC was 0.9537 (95% CI: 0.9282–0.9735), sensitivity was 0.9388 (95% CI: 0.9000–0.9775), specificity was 0.7711 (95% CI: 0.6807–0.8615), and F1 score was 0.9079 (95% CI: 0.8703–0.9401), with corresponding precision and accuracy of 0.8790 and 0.8783, respectively. The confidence intervals of all metrics indicated good stability of its performance estimates. The DeLong test results showed that the AUC differences between AutoML and all compared models were statistically significant. Among them, the differences compared with logistic regression, support vector machine, AdaBoost, and XGBoost were highly significant (all *p* < 0.001).

**Table 3:** Predictive performance of each model in the testing set and DeLong test comparison with AutoML.

Models	PRE	SEN	SPE	ACC	F1	PR-AUC	ROC-AUC	ΔROC-AUC (95% CI)*	DeLong P
LR	0.7228	0.9048	0.3855	0.7174	0.8036	0.7148	0.7575	0.1962 (0.1300, 0.2624)	<0.001
SVM	0.6927	0.966	0.2410	0.7043	0.8068	0.6754	0.7263	0.2274 (0.1602, 0.2946)	<0.001
AdaBoost	0.7831	0.8844	0.5663	0.7696	0.8307	0.7597	0.804	0.1497 (0.0851, 0.2143)	<0.001
XGBoost	0.7812	0.8503	0.5783	0.7522	0.8143	0.8158	0.8319	0.1218 (0.0520, 0.1916)	<0.001
LightGBM	0.7302	0.9388	0.3855	0.7391	0.8214	0.8222	0.8546	0.0991 (0.0105, 0.1877)	0.03
AutoML	**0.8790**	**0.9388**	**0.7711**	**0.8783**	**0.9079**	**0.9400**	**0.9537**	Reference model	—

*ΔAUC = AutoML AUC – Comparison model AUC, 95% CI calculated based on the variance estimate of the DeLong test; this confidence interval directly corresponds to the DeLong *p*-value. Bold data indicate optimal results.

**Table 4:** 95% confidence intervals of the main discriminative metrics for all models in the testing set.

Models	SEN (95% CI)	SPE (95% CI)	F1 (95% CI)	ROC-AUC (95% CI)
LR	0.9048 (0.8573–0.9522)	0.3855 (0.2808–0.4903)	0.8036 (0.7527–0.8491)	0.7575 (0.6890–0.8201)
SVM	0.9660 (0.9367–0.9953)	0.2410 (0.1490–0.3329)	0.8068 (0.7599–0.8491)	0.7263 (0.6521–0.7936)
AdaBoost	0.8844 (0.8327–0.9360)	0.5663 (0.4596–0.6729)	0.8307 (0.7801–0.8754)	0.8040 (0.7426–0.8579)
XGBoost	0.8503 (0.7928–0.9079)	0.5783 (0.4721–0.6845)	0.8143 (0.7600–0.8623)	0.8319 (0.7731–0.8825)
LightGBM	0.9388 (0.9000–0.9775)	0.3855 (0.2808–0.4903)	0.8214 (0.7727–0.8649)	0.8546 (0.7983–0.9025)
AutoML	0.9388 (0.9000–0.9775)	0.7711 (0.6807–0.8615)	0.9079 (0.8703–0.9401)	0.9537 (0.9282–0.9735)

All 95% CIs were calculated based on 2,000 stratified bootstrap resampling iterations.

Decision curve analysis demonstrated that AutoML provided higher net benefit than all other models across a broad risk threshold interval ranging from 1 to 99% (Figure 4E). In terms of probability calibration, the Brier score of AutoML was only 0.103 (Figure 4F), the lowest among the six models. Its 95% confidence interval of the Brier score based on 2,000 bootstrap resampling iterations was 0.082–0.128, which also did not overlap with that of the next best LightGBM model (0.134–0.203), further confirming the statistically significant advantage of AutoML in the accuracy of predicted probabilities.

To further justify the use of SMOTE, we repeated the testing-set evaluation without SMOTE. For the AutoML model, removal of SMOTE increased sensitivity from 0.9388 to 0.9592 but decreased specificity from 0.7711 to 0.6145, accuracy from 0.8783 to 0.8348, F1-score from 0.9079 to 0.8812, and ROC-AUC from 0.9537 to 0.9312; the Brier score also worsened from 0.103 to 0.141. These changes are consistent with a stronger prediction preference for the majority contusion class when the minority non-contusion class is not oversampled. Therefore, SMOTE targeting non-contusion cases, followed by Platt recalibration on the original-distribution validation fold, was retained in the primary analysis.

### 3.4 Sensitivity analysis

**Figure 6:**
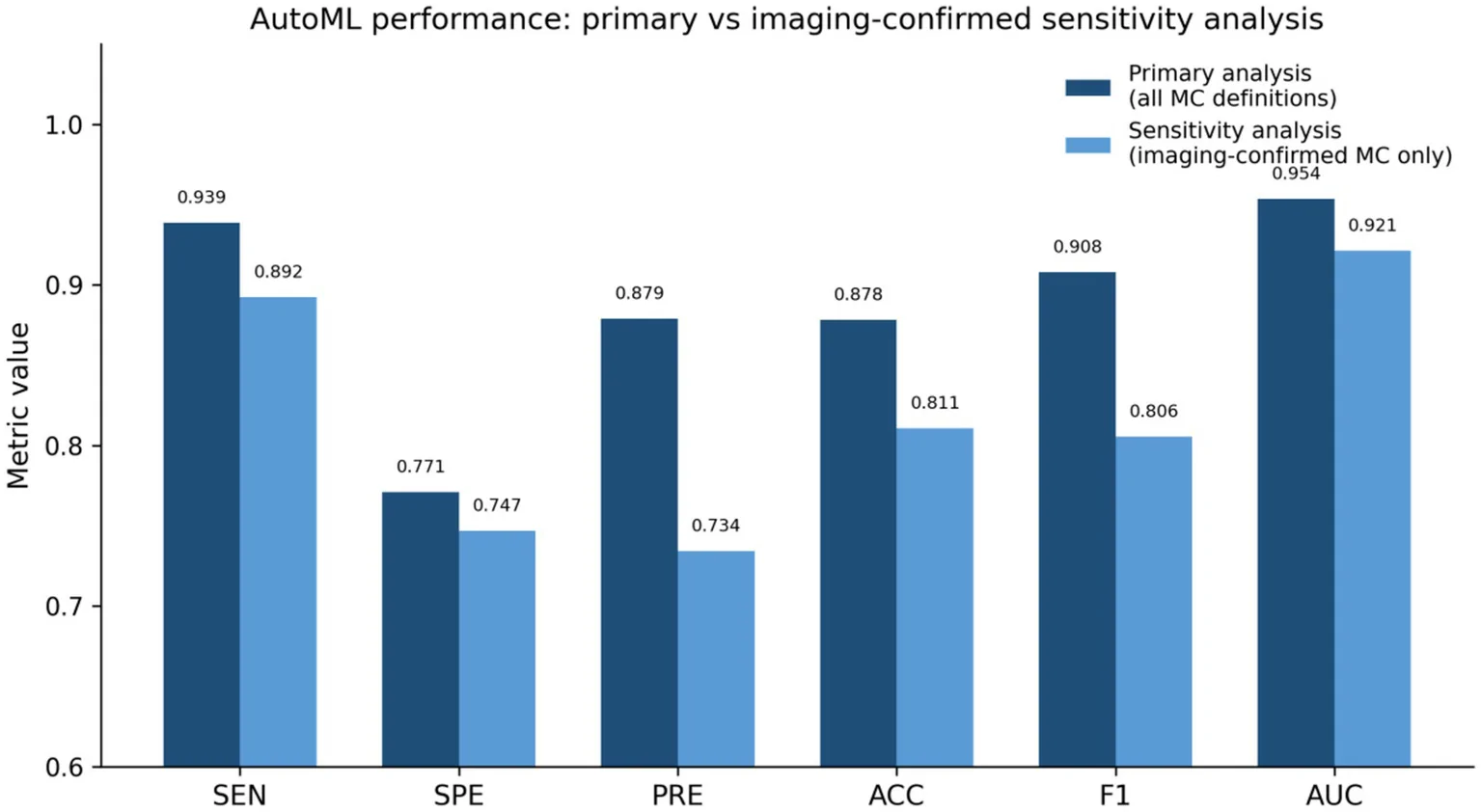
Performance comparison between the main AutoML analysis and the imaging-confirmed sensitivity analysis on the testing set.

Among all 1,038 patients, 268 (25.82%) were confirmed myocardial contusion, 339 (32.66%) were probable myocardial contusion, and 431 (41.52%) were non-myocardial contusion. The completion rates for both electrocardiography and hs-cTnI were 100%; transthoracic echocardiography was completed in 486 cases (46.8%), and contrast-enhanced cardiac MRI was completed in 118 cases (11.4%). In a sensitivity analysis retaining only imaging-confirmed cases and non-contusion cases (test set *n* = 148), AutoML still maintained good discriminative ability (threshold = 0.5; TP = 58, TN = 62, FP = 21, FN = 7; SEN = 0.8923, SPE = 0.7470, PRE = 0.7342, ACC = 0.8108, F1 = 0.8056, AUC = 0.9214). Although slightly decreased compared with the main analysis, it still outperformed the control models, as detailed in Figure 6.

### 3.5 Key determinants analysis

**Figure 7:**
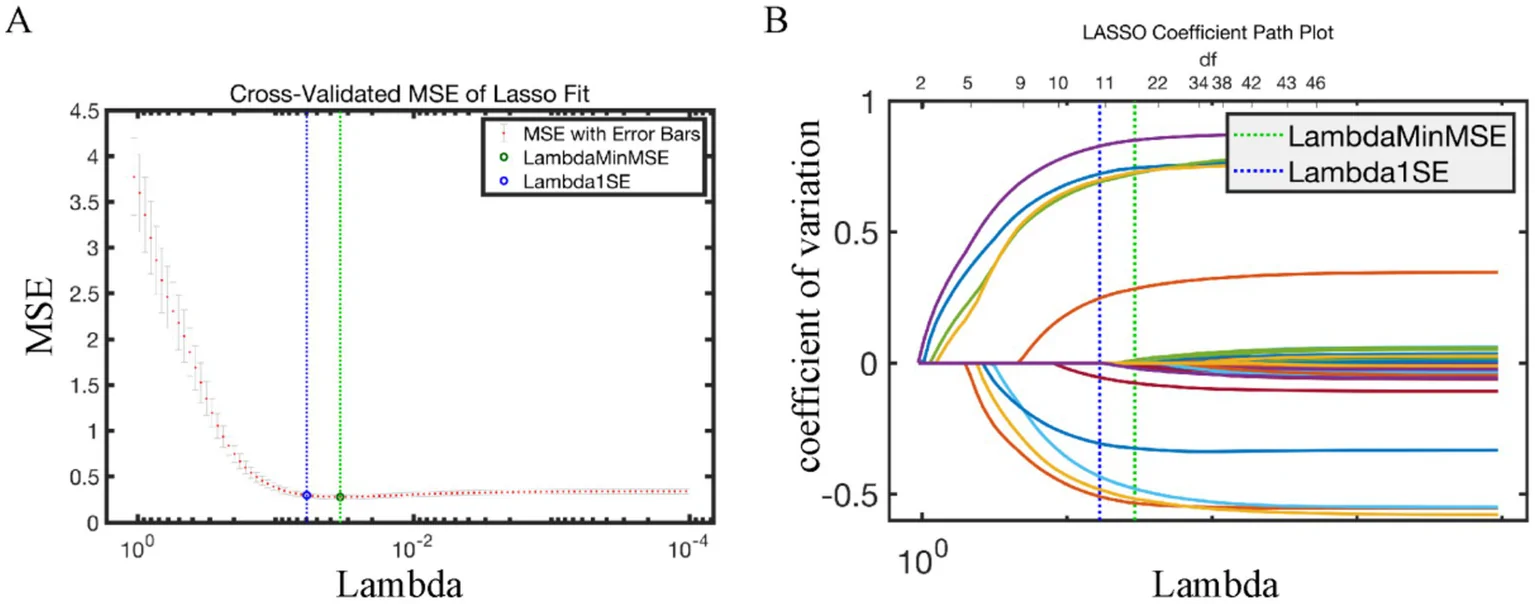
LASSO regression results. **(A)** LASSO trajectory plot; **(B)** LASSO cross-validation fitting plot.

**Figure 8:**
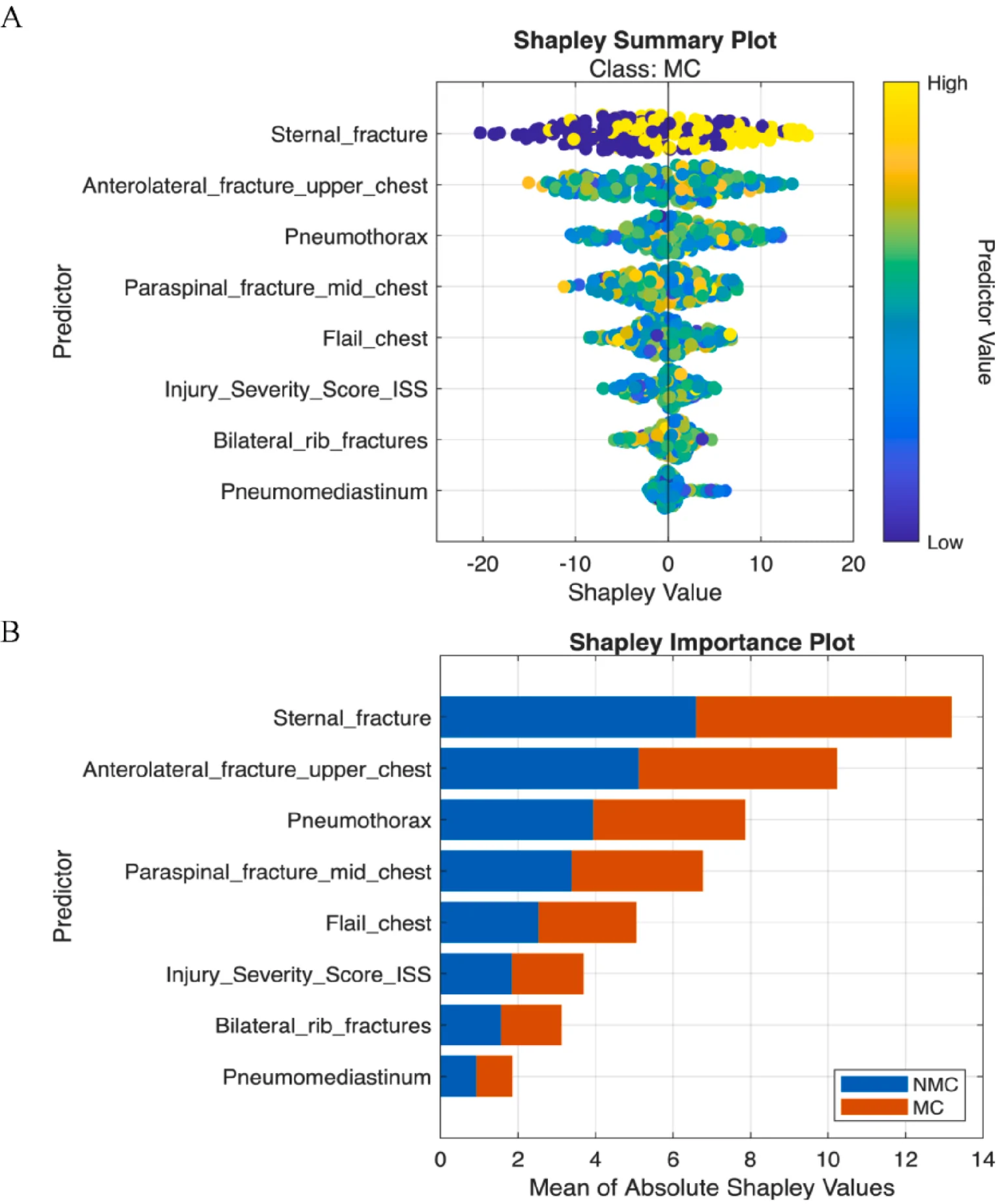
Overall SHAP value comparison of key indicators. **(A)** SHAP summary plot; **(B)** SHAP feature importance plot.

1. LASSO: LASSO regression was used as an independent supplementary consistency analysis to evaluate the stability of the core features identified by AutoML. Under the minimum cross-validation error criterion, LASSO selected 10 variables, including sternal fracture, anterolateral fracture upper chest, pneumothorax, paraspinal fracture mid chest, flail chest, ISS, bilateral rib fracture, pneumomediastinum, hemothorax, and white blood cells (Figure 7). Among them, the first 8 variables were consistent with the 8 core features ultimately selected by AutoML, suggesting that the core AutoML features gained supplementary support within the LASSO regularization framework. Meanwhile, LASSO additionally selected hemothorax and white blood cell count, indicating that the LASSO results were not completely identical to those of AutoML; these two additional variables are only reported as supplementary findings and were not incorporated into the final AutoML prediction model.
2. SHAP analysis: feature importance ranking: Sternum fracture; Anterolateral fracture upper chest; Pneumothorax; Paraspinal fracture mid chest; Flail chest; ISS; Bilateral rib fracture; Pneumomediastinum (Figure 8).

**Figure 9:**
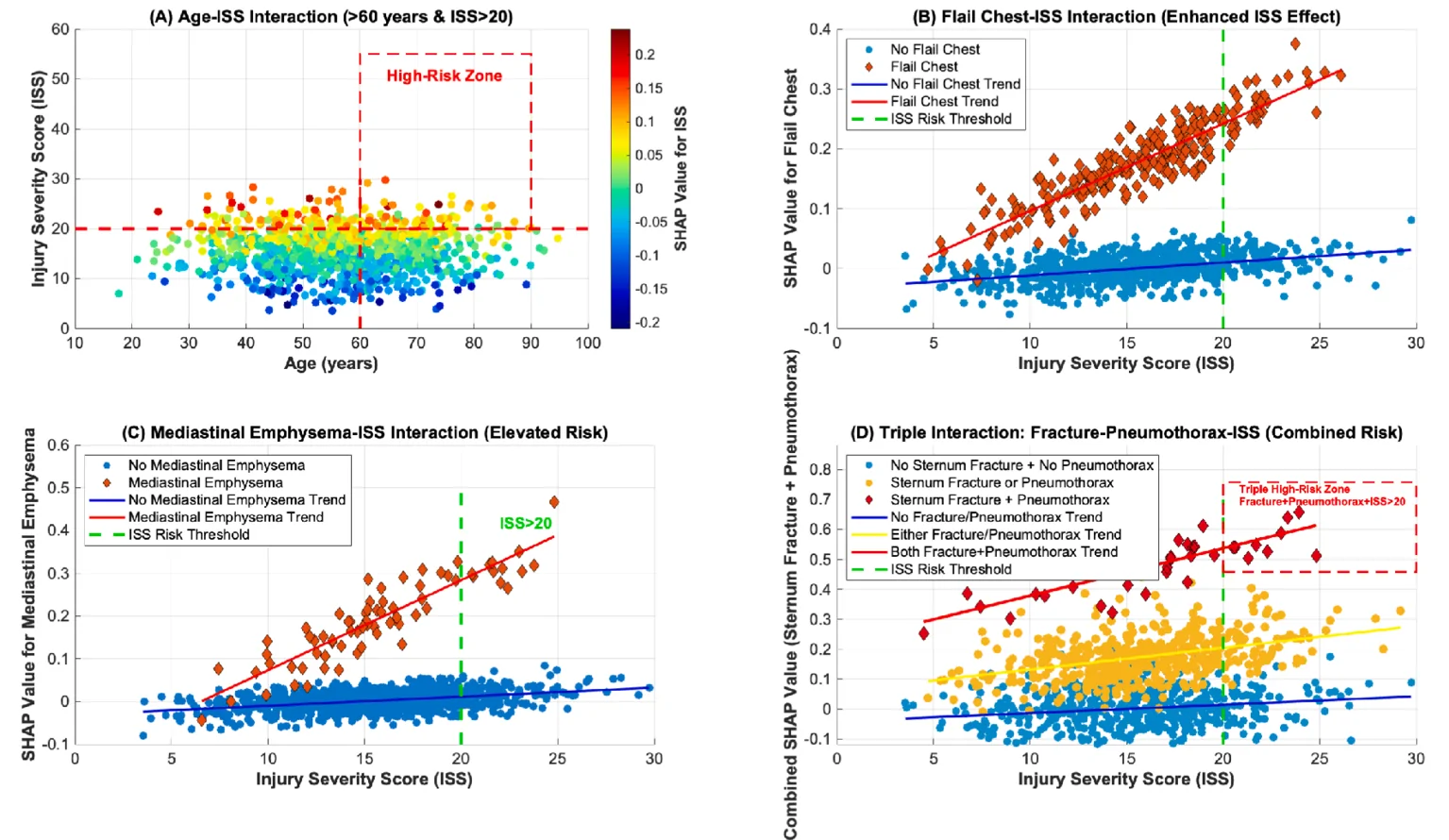
SHAP interaction analysis between key indicators.

SHAP interaction analysis (Figure 9) revealed: Dual high-risk: Age>60 + ISS > 20 (high SHAP values, red) concentrating risk in elderly trauma patients. Synergistic effect: Flail chest amplified ISS impact. Pneumomediastinum: Risk escalated with ISS > 20. Triple high-risk: Sternum fracture + pneumothorax + ISS > 20 (red diamond markers) warranting urgent attention.

### 3.6 Interactive tool

**Figure 10:**
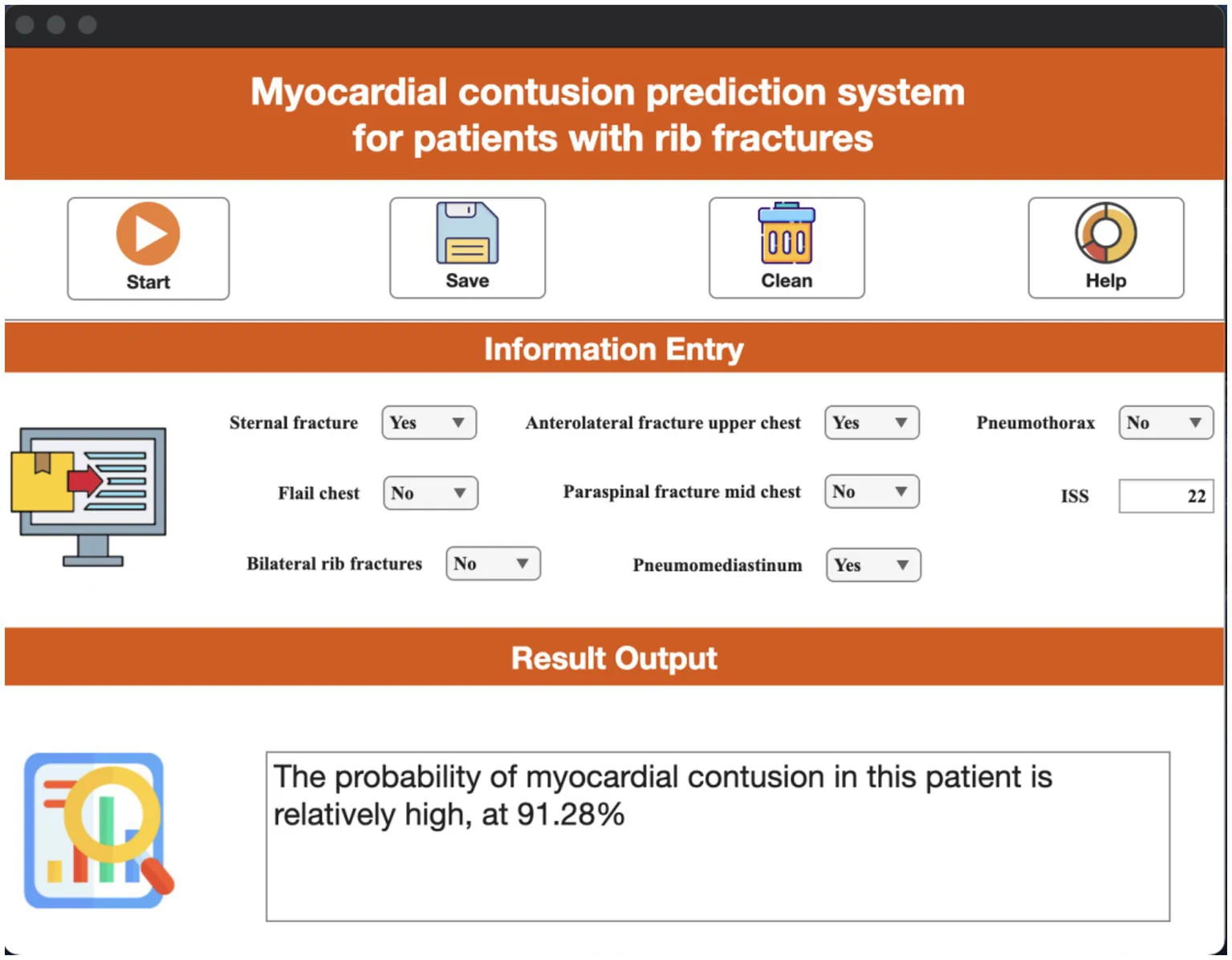
Software system demonstration.

A MATLAB App Designer tool was developed as a prototype to illustrate the potential translation of the prediction model into clinical practice. Through this interface, users can input values for 8 key features to instantly calculate myocardial contusion risk without requiring programming expertise (Figure 10).

## 4 Discussion

This study systematically constructed and validated an automatic machine learning prediction model for myocardial contusion (MC) in rib fracture patients, with its core findings highlighting the clinical value of intelligent technology for optimizing trauma patient risk stratification and guiding early intervention. Given that current clinical practice relies mainly on electrocardiographic changes and traditional serological markers like troponin for early MC diagnosis. However, these anatomical trauma scores do not contain diagnostic elements of myocardial contusion (such as electrical abnormalities or troponin dynamics); in this study, the cardiac AIS code corresponding to myocardial contusion was likewise excluded from the score calculation. Therefore, these variables are mathematically independent as predictive features, yet they still cannot precisely quantify the propensity risk for myocardial contusion. This may lead to severe consequences including malignant arrhythmias, myocardial infarction, or even sudden death (16). The AutoML predictive model developed in this study represents a key effort to address this gap.

In terms of predictive performance, the AutoML model performed outstandingly on both the training and testing sets (training set, threshold = 0.5: SEN = 0.9826, SPE = 0.7874, PRE = 0.8593, ACC = 0.8985, F1 = 0.9168, AUC = 0.9692; testing set, threshold = 0.5: SEN = 0.9388, SPE = 0.7711, PRE = 0.8790, ACC = 0.8783, F1 = 0.9079, AUC = 0.9537), showing clear advantages over traditional models such as LR, SVM, and AdaBoost. To further evaluate the robustness of our findings, we conducted a sensitivity analysis restricted exclusively to imaging-confirmed cases of myocardial contusion. In this conservative scenario, the AutoML model maintained robust performance (AUC = 0.9214, sensitivity = 0.8923, specificity = 0.7470, F1 = 0.8056), which, although slightly attenuated compared with the primary analysis (AUC = 0.9537), remained clinically acceptable and consistently outperformed the comparator models. These findings align with El-Menyar et al.’s (17) conclusions regarding AutoML’s superiority in trauma outcome prediction, though our model uniquely achieves a better sensitivity-specificity balance. Crucially, it maintains robust performance against class distribution shift in the testing set, which holds particular significance for managing class-imbalanced patients in actual clinical scenarios (18, 19).

Regarding feature-outcome correlations, key feature selection revealed intrinsic relationships between anatomical injury localization, biomechanical transmission mechanisms, and overall injury burden. The SHAP explainable framework enabled quantitative analysis of feature contributions while elucidating synergistic effects between anatomical characteristics and overall injury dynamics. LASSO regression validated AutoML’s feature selection by identifying sternum fracture, anterolateral upper thoracic fracture, pneumomediastinum, and bilateral rib fractures as critical risk factors. SHAP global feature importance further quantified the impact of key features: sternal fracture contributed most significantly, which is biologically plausible given its proximity to the heart and the potential for direct transmission of blunt force to the myocardium (10, 20); anterolateral upper thoracic fractures were associated with increased indirect myocardial impact risk, possibly due to high mechanical energy transmission loss characteristics; pneumomediastinum was indicative of severe mediastinal vibration, suggesting that disordered bioenergetic transmission within the mediastinum may represent a key area for future mechanistic investigation in myocardial contusion (21, 22); other significant variables included Injury Severity Score (ISS), pneumothorax, and bilateral rib fractures. Together, these findings suggest a hypothesis-generating framework linking anatomical injury patterns, functional disturbances, and potential metabolic correlates of trauma response (e.g., the association between high ISS and sternal fracture combinations is consistent with efficient transmission of high-impact energy to myocardial tissue), which may inform future studies on high-risk patient identification. Importantly, SHAP dependence analysis revealed two notable feature interactions: (1) An interaction between high ISS (>20) and pneumomediastinum was observed, suggesting that coexisting systemic stress responses and mediastinal pressure fluctuations may constitute clinically relevant superimposed injury patterns (23, 24); (2) An interaction between sternal fracture and pneumothorax was identified, which is consistent with a mechanical pathway where force transmits through a collapsed thorax to the mediastinum, potentially amplifying dual physical damage mechanisms to the myocardium (25, 26). It is important to emphasize that while SHAP analysis provides quantitative insights into feature contributions within the predictive model, it cannot establish causal relationships or biological mechanisms. The identified feature interactions and importance rankings should therefore be regarded as hypothesis-generating observations that require validation through prospective clinical studies and mechanistic experiments.

Several important limitations require further investigation. Firstly, the retrospective design employed uniform inclusion criteria and structured data collection to ensure cohort consistency. However, the absence of a prospective multicenter validation cohort may challenge model robustness against temporal shifts caused by diagnostic equipment upgrades or resuscitation protocol modifications. Another limitation concerns the generalisability of the present findings. Although the study enrolled patients from multiple centers, the validation cohort was drawn from the same regional hospital network and overlapping time period as the training cohort. Thus, this assessment represents geographically limited internal validation rather than a true external validation, and the model performance in independent trauma systems, different clinical settings, or populations with distinct injury patterns remains unknown. Temporal shifts due to changes in diagnostic equipment, clinical management protocols, or case-mix could further challenge model robustness when deployed in future practice. To establish transportability, external validation in geographically and organizationally unconnected trauma centers is essential. Future investigations should incorporate validation using real-world hospital datasets from different healthcare systems, which would strengthen cross-institutional generalizability and evaluate performance across a broader spectrum of injury severities and patient demographics. Second, while representative laboratory indicators and trauma scores were incorporated, the model currently lacks interfaces for specific myocardial injury biomarkers such as troponin kinetics, high-frequency ECG monitoring, or real-time imaging parameters. One important methodological concern relates to the potential for verification bias inherent in the retrospective study design. Since advanced cardiac imaging modalities—including contrast-enhanced cardiac MRI and transthoracic echocardiography—were not uniformly performed on every patient but rather ordered at the discretion of the treating clinicians, it is plausible that these confirmatory examinations were preferentially applied to patients exhibiting higher clinical suspicion of myocardial injury, such as those presenting with overt ECG abnormalities or markedly elevated cardiac troponin levels. This differential verification could have resulted in an overestimation of the true prevalence of myocardial contusion and may have distorted the observed predictor-outcome associations. We fully acknowledge this limitation, which cannot be completely eliminated in a retrospective analysis. Future prospective validation studies should implement a standardized protocol in which all enrolled patients undergo protocolized cardiac imaging assessment, thereby ensuring complete and non-differential verification of the outcome and enabling unbiased estimation of both disease prevalence and model performance. Third, the interactive tool requires functional expansion. While the MATLAB-based interface enables intuitive data input and prediction output, automated integration with hospital electronic medical record systems remains unrealized. Future studies should develop automated tools for real-time heterogeneous data acquisition and dynamic model iteration while ensuring interpretable outputs. Additional validation using real-world hospital datasets would strengthen cross-institutional generalizability, enabling expansion into an intelligent dynamic warning network integrating lab reports, imaging findings, and pathophysiological alerts. Finally, the proposed anatomical localization and trauma dynamics hypotheses require prospective endpoint validation. It should be emphasized that the present study focuses on model development and internal validation; the clinical utility, workflow integration, and generalizability of the proposed tool remain to be rigorously tested in prospective clinical settings. Exploratory directions for future studies may include biomechanical simulations linking chest wall injury patterns to myocardial strain responses and, eventually, molecular imaging correlatives. These represent distinct and ambitious long-term aims that require dedicated experimental designs and independent validation beyond the scope of the current prediction model.

## 5 Conclusion

In summary, this study has preliminarily established an intelligent early-warning system for myocardial contusion risk in rib fracture patients through comprehensive clinical data mining, advanced algorithm optimization, multi-dimensional feature selection validation, and human-centered multi-unit collaborative design. The SHAP explainable platform has enabled refined multimodal feature contribution analysis, establishing a visualized framework for exploring potential high-risk factor combinations that warrant further investigation. It is important to emphasize that SHAP analysis quantifies feature contributions within the predictive model but cannot prove biological mechanisms, causal relationships, or mechanical pathways. These exploratory findings therefore generate hypotheses regarding possible associations between anatomical positioning and trauma-induced cardiac responses, which require dedicated prospective studies and experimental validation to establish causality and elucidate any underlying molecular mechanisms. Future research should enhance model robustness through prospective multicenter data validation. Integration of high-precision force-field simulations with molecular imaging biomarker chains will create cross-domain recognition mapping. Future research may contribute, in the long term, to a more integrated clinical decision-support framework encompassing early recognition, risk stratification, and mechanism-informed prevention. However, substantial additional work—including prospective validation, multimodal data integration, and real-world implementation studies—is required before such a vision can be approached.

## Data Availability

The raw data supporting the conclusions of this article will be made available by the authors, without undue reservation.

## References

[1] Mehta H Gheith Z Amin S Acharya P Daon E Downey P . Trends of hospitalizations and in-hospital outcomes for traumatic cardiac injury in United States. Kans J Med. (2024) 17:45–50. doi: 10.17161/kjm.vol17.21442 38859990 PMC11164420

[2] Gao JM Li H Wei GB Liu CP du DY Kong LW . Blunt cardiac injury: a single-center 15-year experience. Am Surg. (2020) 86:354–61. doi: 10.1177/000313482008600432, 32391760

[3] Van Lieshout EMM Verhofstad MHJ Van Silfhout DJT Dubois EA. Diagnostic approach for myocardial contusion: a retrospective evaluation of patient data and review of the literature. Eur J Trauma Emerg Surg. (2021) 47:1259–72. doi: 10.1007/s00068-020-01305-4, 31982920 PMC8321993

[4] Morley EJ English B Cohen DB Paolo WF. Blunt cardiac injury: emergency department diagnosis and management. Emerg Med Pract. (2019) 21:1–20. 30794369

[5] Nathwani JN Baucom MR Salvator A Makley AT Tsuei BJ Droege CA . Evaluating the utility of high sensitivity troponin in blunt cardiac injury. J Surg Res. (2023) 281:104–11. doi: 10.1016/j.jss.2022.08.030, 36152398

[6] Kalbitz M Pressmar J Stecher J Weber B Weiss M Schwarz S . The role of troponin in blunt cardiac injury after multiple trauma in humans. World J Surg. (2017) 41:162–9. doi: 10.1007/s00268-016-3650-7, 27501709

[7] Grigorian A Milliken J Livingston JK Spencer D Gabriel V Schubl SD . National risk factors for blunt cardiac injury: hemopneumothorax is the strongest predictor. Am J Surg. (2019) 217:639–42. doi: 10.1016/j.amjsurg.2018.07043 30060913

[8] Burrell AJ Kaye DM Fitzgerald MC Cooper DJ Hare JL Costello BT . Cardiac magnetic resonance imaging in suspected blunt cardiac injury: a prospective, pilot, cohort study. Injury. (2017) 48:1013–9. doi: 10.1016/j.injury.2017.02.025, 28318537

[9] Hammer MM Raptis DA Cummings KW Mellnick VM Bhalla S Schuerer DJ . Imaging in blunt cardiac injury: computed tomographic findings in cardiac contusion and associated injuries. Injury. (2016) 47:1025–30. doi: 10.1016/j.injury.2015.11.008, 26646729

[10] Kyriazidis IP Jakob DA Vargas JAH Franco OH Degiannis E Dorn P . Accuracy of diagnostic tests in cardiac injury after blunt chest trauma: a systematic review and meta-analysis. World J Emerg Surg. (2023) 18:36. doi: 10.1186/s13017-023-00504-9, 37245048 PMC10225099

[11] Emigh B Grigorian A Dilday J Condon F Nahmias J Schellenberg M . Risk factors and outcomes in pediatric blunt cardiac injuries. Pediatr Surg Int. (2023) 39:195. doi: 10.1007/s00383-023-05478-y, 37160488

[12] Hanschen M Kanz KG Kirchhoff C Khalil PN Wierer M van Griensven M . Blunt cardiac injury in the severely injured-a retrospective multicentre study. PLoS One. (2015) 10:e0131362. doi: 10.1371/journal.pone.0131362, 26136126 PMC4489656

[13] Dua A McMaster J Desai PJ Desai SS Kuy S Mata M . The association between blunt cardiac injury and isolated sternal fracture. Cardiol Res Pract. (2014) 2014:629687. doi: 10.1155/2014/629687 24653859 PMC3933392

[14] Ishida K Katayama Y Kitamura T Hirose T Ojima M Nakao S . Factors associated with cardiac/pericardial injury among blunt injury patients: a nationwide study in Japan. J Clin Med. (2022) 11:4534. doi: 10.3390/jcm11154534, 35956149 PMC9369737

[15] Sandoval Y Apple FS Mahler SA Body R Collinson PO Jaffe AS . High-sensitivity cardiac troponin and the 2021 AHA/ACC/ASE/CHEST/SAEM/SCCT/SCMR guidelines for the evaluation and diagnosis of acute CHEST pain. Circulation. (2022) 146:569–81. doi: 10.1161/CIRCULATIONAHA.122.059678, 35775423

[16] Jung LY Rhee KS. Delayed complete heart block in blunt cardiac injury (myocardial contusion). Eur Heart J Cardiovasc Imaging. (2023) 24:e203. doi: 10.1093/ehjci/jead105, 37195884

[17] El-Menyar A Naduvilekandy M Asim M Rizoli S Al-Thani H. Machine learning models predict triage levels, massive transfusion protocol activation, and mortality in trauma utilizing patients hemodynamics on admission. Comput Biol Med. (2024) 179:108880. doi: 10.1016/j.compbiomed.2024.108880, 39018880

[18] Değer SU. A study on heart data analysis and prediction using advanced machine learning methods. Comput Biol Med. (2025) 192:110308. doi: 10.1016/j.compbiomed.2025.110308, 40359679

[19] Farzaneh N Williamson CA Gryak J Najarian K. A hierarchical expert-guided machine learning framework for clinical decision support systems: an application to traumatic brain injury prognostication. NPJ Digit Med. (2021) 4:78. doi: 10.1038/s41746-021-00445-0, 33963275 PMC8105342

[20] Lynch SD Taylor SL Greene KA Devane KS Weaver AA. Characterizing thoracic morphology variation to develop representative 3D models for applications in chest trauma. Comput Biol Med. (2023) 163:107211. doi: 10.1016/j.compbiomed.2023.107211, 37390760

[21] Li Z Xu H Fan F. Approach to mediastinal fine needle aspiration cytology. Adv Anat Pathol. (2022) 29:337–48. doi: 10.1097/PAP.0000000000000355, 35838636

[22] Sztulman L Ritter A de Rosa R Pfeiffer V Leppik L Busse LC . Cardiac damage after polytrauma: the role of systematic transthoracic echocardiography-a pilot study. World J Emerg Surg. (2025) 20:21. doi: 10.1186/s13017-025-00596-5, 40069898 PMC11895250

[23] Latifi A Wang D Backer ED Madisi N Chopra A Kapp CM . Pleural manometry in pneumothorax: evaluating tension physiology and predicting outcomes. Chest. (2025) 10:S0012-3692(25)05492-3. doi: 10.1016/j.chest.2025.09.121 PMC12925486 41076067

[24] Coccolini F Cremonini C Moore EE Civil I Balogh Z Leppaniemi A . Thoracic trauma WSES-AAST guidelines. World J Emerg Surg. (2025) 20:78. doi: 10.1186/s13017-025-00651-1, 41094688 PMC12522690

[25] Caragounis EC Xiao Y Granhed H. Mechanism of injury, injury patterns and associated injuries in patients operated for chest wall trauma. Eur J Trauma Emerg Surg. (2021) 47:929–38. doi: 10.1007/s00068-019-01119-z, 30953111 PMC8319693

[26] Gupta B Singh Y Bagaria D Nagarajappa A. Comprehensive management of the patient with traumatic cardiac injury. Anesth Analg. (2023) 136:877–93. doi: 10.1213/ANE.0000000000006380, 37058724

