# Supplementary material for: Development and validation of an automatic machine learning-based myocardial contusion prediction model in rib fracture patients: a retrospective multicenter cohort study
Source: Front Med (Lausanne). 2026 Sep 3;13:1855699. doi: 10.3389/fmed.2026.1855699 (PMC13581564; doi:10.3389/fmed.2026.1855699)
Supplement: Supplementary file 1 [file Data_sheet_2.DOCX]

**Algorithm Optimization Performance**

The enhanced ICPO algorithm outperformed CPO, WOA, GWO, PSO, GA, GA-PSO, and GA-ACO using all 12 CEC2022 benchmark functions. Parameters included: Variable dimension: 10; Population size: 30; Maximum iterations: 500; Independent runs: 30. Box plots of optimization results (**Fig. S1A**) demonstrated ICPO’s superior stability across most functions compared to baseline CPO and other algorithms. Convergence curves (**Fig. S2B**) revealed ICPO achieved faster convergence speed and minimal local optima risk during iterations. These results validated ICPO’s global optimization efficacy .

**Fig. S1: Modified Swarm Intelligence Algorithm Simulation Test**

Note: A: Box plots of optimization results after 30 independent runs of each algorithm on CEC2022 test functions, illustrating optimization stability and robustness; B: Convergence curves during optimization, reflecting convergence speed and ability to avoid local optima
