# Supplementary material for: Development and validation of an automatic machine learning-based myocardial contusion prediction model in rib fracture patients: a retrospective multicenter cohort study
Source: Front Med (Lausanne). 2026 Sep 3;13:1855699. doi: 10.3389/fmed.2026.1855699 (PMC13581564; doi:10.3389/fmed.2026.1855699)
Supplement: Supplementary file 2 [file Table_1.DOCX]

**Pseudocode: Core Optimization Workflow of the ICPO-Based AutoML Framework**

%% 1. Data Preparation and Global Partitioning

load patient_data.mat; % Load feature matrix X and label vector y

rng(42); % Fix random seed for reproducibility

cv_part = cvpartition(y, 'Holdout', 0.2); % Set aside 20% of the data as the independent test set

X_train = X(training(cv_part), :); y_train = y(training(cv_part));

X_test = X(test(cv_part), :); y_test = y(test(cv_part));

K = 5; % Number of inner cross-validation folds

p = size(X_train, 2); % Total number of features

dim_features = p; % Dimensionality of the feature mask vector

dim_hyperparams = 3; % Number of hyperparameters to optimize (e.g., learning rate, max depth, num leaves)

dim_total = dim_features + dim_hyperparams; % Total dimensionality of each ICPO particle

%% 2. Define Search-Space Boundaries

% The first p elements correspond to feature masks (continuous [0,1], then discretized at 0.5).

lb = zeros(1, dim_total);

ub = ones(1, dim_total);

% Remaining three elements are hyperparameters; rescale them to their actual ranges as needed.

% For instance:

% ub(p+1) = 0.5; % Upper bound for learning rate

% ub(p+2) = 15; % Upper bound for max_depth (rounded to integer inside the pipeline)

% lb(p+2) = 3;

% ... similar definitions

%% 3. Nested Cross-Validation and ICPO Main Loop

cv_indices = crossvalind('Kfold', y_train, K); % Generate fold assignments for inner CV

best_fitness_history = zeros(K,1); % Store best fitness (1-AUC) for each fold

best_solutions = cell(K,1); % Store best position vectors

for fold = 1 : K

% ---------- 3.1 Split into inner training and validation sets ----------

val_idx = (cv_indices == fold);

tr_idx = ~val_idx;

X_inner_tr = X_train(tr_idx, :); % Inner raw training features

y_inner_tr = y_train(tr_idx);

X_val = X_train(val_idx, :); % Inner raw validation features

y_val = y_train(val_idx); % Validation labels kept untouched

% ---------- 3.2 Apply SMOTE only to the inner training set ----------

% SMOTE synthesizes new minority-class samples based on X_inner_tr and y_inner_tr.

% The validation set (X_val, y_val) is never involved in this step,

% guaranteeing zero information leakage.

[X_inner_tr_smote, y_inner_tr_smote] = SMOTE(X_inner_tr, y_inner_tr);

% ---------- 3.3 Construct the fitness (objective) function ----------

% The function evaluate_pipeline takes a particle vector x, decodes it into

% a feature mask and hyperparameters, trains a LightGBM model on the SMOTE-

% balanced inner training set, evaluates AUC on the original inner validation

% set, and returns 1 - AUC as the loss to be minimized.

% If the decoded feature mask contains all zeros, a large penalty is returned

% to force the selection of at least one feature.

fitness_func = @(x) evaluate_pipeline(x, ...

X_inner_tr_smote, y_inner_tr_smote, X_val, y_val, dim_features);

% ---------- 3.4 Invoke the Improved Crested Porcupine Optimization (ICPO) ----------

% The original Crested Porcupine Optimization (CPO) is a swarm intelligence

% algorithm inspired by the collective foraging and anti-predation behaviors

% of crested porcupines. It simulates four defense/foraging phases (odor

% diffusion, sound intimidation, quill defense, and back attack), uses

% Lévy flight for global exploration, and follows the global best for

% local exploitation.

%

% ICPO improves upon CPO through two key modifications:

% 1) Chaotic mapping is employed during population initialization to enhance

% diversity and ergodicity of the initial solutions.

% 2) A dynamic Lévy flight strategy adaptively adjusts step sizes according

% to iteration progress, achieving a better balance between exploration

% and exploitation.

% The effectiveness of these improvements has been validated with the

% CEC2022 benchmark suite.

pop_size = 30; % Population size

max_iter = 100; % Maximum number of iterations

% Execute ICPO (the chaotic initialization and dynamic Lévy flight are

% encapsulated inside the function).

[best_x, best_fitness] = ICPO(fitness_func, dim_total, lb, ub, ...

pop_size, max_iter);

% ---------- 3.5 Save the best solution and its performance for this fold ----------

best_solutions{fold} = best_x;

best_fitness_history(fold) = best_fitness; % best_fitness = 1 - AUC

end

%% 4. Aggregate Results from K Folds and Determine Final Feature Set and Hyperparameters

% 4.1 Vote for features: a feature is retained if selected in ≥50% of the folds

feature_masks = zeros(K, dim_features);

for fold = 1:K

feature_masks(fold, :) = (best_solutions{fold}(1:dim_features) >= 0.5);

end

vote_ratio = mean(feature_masks, 1);

final_feature_idx = find(vote_ratio >= 0.5); % Robust feature subset

% 4.2 Select hyperparameters from the fold with the best fitness (lowest 1-AUC)

[~, best_fold] = min(best_fitness_history);

final_hyperparams = best_solutions{best_fold}(dim_features+1 : end);

%% 5. Train Final Model on the Full Training Set and Evaluate on the Independent Test Set

% 5.1 Extract final features from the training set, then apply SMOTE

X_train_final_feat = X_train(:, final_feature_idx);

[X_train_balanced, y_train_balanced] = SMOTE(X_train_final_feat, y_train);

% 5.2 Train the final LightGBM model (actual parameter unpacking is omitted for brevity)

final_model = train_lightgbm(X_train_balanced, y_train_balanced, final_hyperparams);

% 5.3 Predict on the completely untouched independent test set

X_test_final_feat = X_test(:, final_feature_idx);

y_test_pred = predict(final_model, X_test_final_feat);

% Compute test-set performance metrics

[AUC, SEN, SPE, PRE, F1] = compute_metrics(y_test, y_test_pred);

fprintf('Test set performance -> AUC: %.4f, SEN: %.4f, SPE: %.4f, F1: %.4f\n', ...

AUC, SEN, SPE, F1);

%% Helper Function evaluate_pipeline (logical flow described in comments)

% function loss = evaluate_pipeline(x, X_tr_smote, y_tr_smote, X_val, y_val, n_feat)

% % 1. Decode feature mask: mask = (x(1:n_feat) >= 0.5);

% % 2. If mask contains no true elements, return inf as a penalty.

% % 3. Reduce X_tr_smote and X_val to the selected features.

% % 4. Decode hyperparameters: map continuous values in x(n_feat+1:end) to

% % actual LightGBM parameters (e.g., learning rate mapped linearly,

% % max_depth linearly scaled and rounded).

% % 5. Train a LightGBM model on the reduced, SMOTE-balanced inner training set

% % using the decoded hyperparameters.

% % 6. Compute AUC on the original (unresampled) validation set X_val.

% % 7. Set loss = 1 - AUC, which ICPO minimizes.

% end

Note: The pseudocode presented here is intended solely to illustrate the general workflow of the ICPO AutoML framework and employs a generic data-splitting configuration for demonstrative purposes. This pseudocode does not reflect the actual cohort partitioning used in this study. The actual dataset division strictly follows the procedure described in the main manuscript (Methods section).
